# A Novel L-Asparaginase from Hyperthermophilic Archaeon *Thermococcus sibiricus*: Heterologous Expression and Characterization for Biotechnology Application

**DOI:** 10.3390/ijms22189894

**Published:** 2021-09-13

**Authors:** Maria Dumina, Alexander Zhgun, Marina Pokrovskaya, Svetlana Aleksandrova, Dmitry Zhdanov, Nikolay Sokolov, Michael El’darov

**Affiliations:** 1 Group of Fungal Genetic Engineering, Federal Research Center “Fundamentals of Biotechnology” of the Russian Academy of Sciences, 117312 Moscow, Russia; zzhgun@mail.ru; 2 Laboratory of Medical Biotechnology, Institute of Biomedical Chemistry, 119121 Moscow, Russia; ivan1190@yandex.ru (M.P.); v-aleksandrov@yandex.ru (S.A.); zhdanovdd@gmail.com (D.Z.); sokolov2144@yandex.ru (N.S.)

**Keywords:** L-asparaginase, hyperthermophile, heterologous expression, biochemical properties, kinetic characteristics, cytotoxic activity

## Abstract

L-asparaginase (L-ASNase) is a vital enzyme with a broad range of applications in medicine and food industry. Drawbacks of current commercial L-ASNases stimulate the search for better-producing sources of the enzyme, and extremophiles are especially attractive in this view. In this study, a novel L-asparaginase originating from the hyperthermophilic archaeon *Thermococcus sibiricus* (TsA) was expressed in *Escherichia coli*, purified and characterized. The enzyme is optimally active at 90 °C and pH 9.0 with a specific activity of 2164 U/mg towards L-asparagine. Kinetic parameters K_M_ and V_max_ for the enzyme are 2.8 mM and 1200 µM/min, respectively. TsA is stable in urea solutions 0–6 M and displays no significant changes of the activity in the presence of metal ions Ni^2+^, Cu^2+^, Mg^2+^, Zn^2+^ and Ca^2+^ and EDTA added in concentrations 1 and 10 mmol/L except for Fe^3+^. The enzyme retains 86% of its initial activity after 20 min incubation at 90 °C, which should be enough to reduce acrylamide formation in foods processed at elevated temperatures. TsA displays strong cytotoxic activity toward cancer cell lines K562, A549 and Sk-Br-3, while normal human fibroblasts WI-38 are almost unsensitive to it. The enzyme seems to be a promising candidate for further investigation and biotechnology application.

## 1. Introduction

L-asparaginase (EC 3.5.1.1; L-asparagine amidohydrolase) (L-ASNase) is a vital enzyme that catalyzes the conversion of L-asparagine amino acid (L-Asn) to L-aspartic acid and ammonia [1]. It has been widely investigated since 1967 with respect to its promising anticancer activity [2].

Unlike normal cells, tumor cells, more specifically leukemia cells, have little or no asparagine synthetase enzyme and require exogenous asparagine to maintain their rapid malignant growth [3,4]. Therefore, the anticancer activity of L-ASNase is due to its depleting effect on the concentration of L-Asn in the extracellular fluid. A diminished level of L-Asn and the inability of tumor cells—in particular susceptible leukemic cells—to synthesize their own L-Asn leads to the inhibition of protein synthesis, cell cycle arrest in the G1 phase and ultimately apoptosis in leukemic cells [5,6].

Recent advances in intensive chemotherapy including L-ASNase treatment, as well as adequate supportive care, have made significant improvements in the treatment of acute lymphoblastic leukemia (ALL) with a cure rate among children of approximately 80% [4,7,8,9]. The reported clinical data show that the enzyme is safe and effective in the treatment of younger adults with ALL with response rates in the frontline setting ranging from 78% to 96%, compared to most trials showing a 4-year overall survival of 50% or better [10].

Currently, three L-ASNase formulations are widely used for the treatment of ALL, namely native *Escherichia coli* asparaginase (Elspar (Merck & Co, Inc., West Point, PA, USA), its PEGylated form (Oncaspar (Enzon Pharmaceuticals Inc., Cranford, NJ, USA) and Erwinase from cultures of *Erwinia chrysanthemi * (Ipsen-Speywood Pharmaceuticals Ltd, Maidenhead, UK) [11,12]. However, these enzymes have drawbacks in that they exhibit low substrate specificity and high glutaminase activity, the latter of which can cause liver dysfunction, pancreatitis, leucopenia, neurological seizures and coagulation abnormalities that can lead to intracranial thrombosis or hemorrhages [13,14].

The application of L-ASNase formulations is also limited by immune system response and antibody production against foreign L-ASNase [15,16]. Anti-asparaginase antibodies are responsible for major toxicity and resistance to L-ASNase therapy [11].

Among the other main restrictions of current commercial L-ASNase are low stability, shorter duration of activity, rapid plasma clearance and multiple injections required to maintain the therapeutic level [5,11,12].

The application in medicine is the original and main but not the only field of use of L-ASNase. The enzyme is used in the food industry as a processing agent to reduce the acrylamide levels in commercial fried foods, maintaining their characteristics (color, flavor, texture, security, etc.) [17,18]. Actually, L-ASNase catalyzes the hydrolysis of L-Asn, not allowing the reaction of reducing sugars with this amino acid for the generation of acrylamide classified as “reasonably anticipated to be a human carcinogen” [19].

The relevance of L-ASNase is not only limited to its use as an anticancer drug and food processing agent. Developed L-ASNase-based biosensors allow monitoring L-Asn levels. Its determination is important both in biochemistry and food chemistry [20,21,22,23].

However, the application of the enzyme in the last-mentioned fields is also limited by the low stability of current commercial L-ASNases. In particular, temperatures in the food industry shoot up to 120 °C or even beyond, resulting in a relatively rapid rate of loss of enzyme activity of L-ASNase from *Aspergillus* species, widely used in baking industries. These enzymes are thermolabile and active only at a narrow range of pH [24,25,26].

L-ASNases are widespread in nature. The enzyme was found in various organisms including animals, plant cells, yeast, fungi and bacteria. L-ASNases derived from various sources show different properties and, in particular, do not share antigenic cross-reactivity [1,12,15,27,28,29,30]. In summary, in view of (i) a great significance of the enzyme, wide range of its potential application and huge market demand and (ii) disadvantages of current commercial enzyme preparations, the industry is still in the search of a better-producing source in terms of high yield and improved characteristics, such as improved stability, low immunogenicity, lower glutaminase activity and high substrate affinity.

Currently, many microbial species, from archaeal, bacterial and eukaryotic sources, have been screened for L-ASNase production [25,31,32,33,34]. In particular, extremophiles have been reported for their potential to produce L-ASNases with extraordinary properties. In recent decades, extremophilic organisms became a valuable source of novel enzymes with superior performances in different areas of biotechnology, food industry and medicine [35,36,37,38,39,40,41].

L-ASNases of extremophilic origin have been already reported from *Thermococcus kodakarensis* [24,34,42], *Thermococcus zilligii* [43], *Thermococcus gammatolerans* [44], *Pyrococcus yayanosii* CH1 [45], *Pyrococcus furiosus* [33,46,47,48], *Pyrococcus horikoshii* [49], *Archaeoglobus fulgidus* [23] and *Pyrobaculum calidifontis* [50], living under extremely high temperatures.

In this study, we describe a novel L-ASNase derived from *Thermococcus sibiricus*.

*T. sibiricus* is a hyperthermophilic anaerobic archaeon isolated from a well of the never-flooded oil-bearing Jurassic horizon of the high-temperature Samotlor oil reservoir (Western Siberia, temperature 60–84 °C) [51]. The sampling site had a temperature of 84 °C and was located at a depth of 2350 m.

The present study reveals cloning, expression, purification and characterization of thermophilic L-ASNase from *T. sibiricus* (TsA). The isolated enzyme, TsA, was assessed for thermal stability for possible application as a food additive and cytotoxic activity for future medical use.

The main properties of novel L-ASNase from *T. sibiricus* are presented in comparison with previously described L-ASNases from other hyperthermophiles, first of all, the closely related members of *Thermococcus* and *Pyrococcus* spp. Our data indicate that L-ASNase from *T. sibiricus* displays novel characteristics and is a promising enzyme for further investigation and biotechnological implementations.

## 2. Results

### 2.1. Identification and Sequence Comparison of TsA

*T. sibiricus* is an archaeon hyperthermophile whose whole genomic sequence has been completed and deposited as GenBank accession number NC_012883 [52,53]. According to the genome information, there is a putative gene TSIB_RS08165 (*tsA*_wt), predicted to encode L-ASNase (TsA) (GenBank accession No. WP_015849943.1).

TSIB_RS08165 gene contained an intact open reading frame of 993 bp and a termination codon, encoding a protein of 331 amino acids.

Amino acid sequence comparison showed that TsA displayed homology with the archaeon L-asparaginases from *Thermococcus litoralis* (GenBank accession No. WP_004066133) at a level of 77%, *Thermococcus zilligii* (GenBank accession No. WP_010478656) at a level of 62%, *Thermococcus gammatolerans* (GenBank accession No. WP_015859055) at a level of 61% and well-characterized L-ASNase originating from *Thermococcus kodakarensis* (WP_011250607) at a level of 63% [34,42].

Comparison of TsA with homologs derived from the members of *Pyrococcus* sp. showed sequence identity with L-ASNase from *Pyrococcus yayanosii* (GenBank accession No. WP_013906452) at a level of 62% and with those from *Pyrococcus furiosus* (GenBank accession No. WP_011013191) and *Pyrococcus horikoshii* (GenBank accession No. WP_010884185) at a level of 56%.

As previously reported, the level of homology between L-ASNases of archaeal and nonarchaeal origin is rather low [43,54]. The identity of TsA and well-characterized mesophilic bacterial L-ASNases from *Pectobacterium atrosepticum* (*Erwinia carotovora* subsp. atroseptica) (GenBank accession No. AAP92666.3) [55,56] and *Rhodospirillum rubrum* (GenBank accession No. QXG80441.1) [57,58,59] is about 27% and 33%, respectively. The identity between TsA and L-ASNases, FDA-approved for use as antileukemic agents, from *E. coli* (UniProtKB accession number P00805, marketed under the brand name Elspar, GenBank accession No. AAA23445.1) and *Erwinia chrysanthemi* (*Dickeya chrysanthemi*, *Pectobacterium chrysanthemi*) (UniProtKB accession number P06608, marketed under the brand name Erwinaze, GenBank accession No. AAS67028.1) is also low, only 28.5% and 29.6%, respectively.

**Figure 1:**
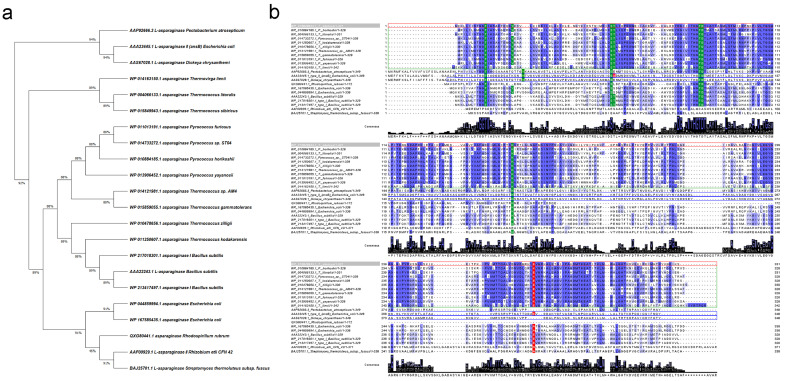
Comparison of L-asparaginase (L-ASNase) from *T. sibiricus* (TsA) with selected L-ASNases of thermophilic and mesophilic origin. (**a**) Molecular phylogenetic analysis of selected L-ASNases by maximum likelihood method. The analysis involved 22 amino acid sequences. There were a total of 371 positions in the final dataset. Evolutionary analyses were conducted in MEGA X [60]. (**b**) Amino acid sequence comparison of L-ASNase *T. sibiricus* (WP_015849943, marked with red box) with its homologs from thermophiles *P. horikoshii* (WP_010884185), *T. litoralis* (WP_004066133), *Pyrococcus* sp. ST04 (WP_014733272), *T. kodakarensis* (WP_011250607), *T. zilligii* (WP_010478656), *Thermococcus* sp. AM4 (WP_014121981), *T. gammatolerans* (WP_015859055), *P. furiosus* (WP_011013191), *P. yayanosii* (WP_013906452), *T. lienii* (WP_014163150) and *Streptomyces thermoluteus* subsp. fuscus (BAJ25701.1) and mesophiles *P. atrosepticum* (AAP92666.3), *E. coli* (type II—AAA23445.1, type I—WP_044859994.1, WP_167585435.1), *D. chrysanthemi* (AAS67028.1), *Bacillus subtilis* (AAA22243.1, type I—WP_217018301.1, WP_213417497.1), *R. rubrum* (QXG80441.1) and *Rhizobium etli* CFN 42 (AAF00929.1). Identical amino acid residues are marked by blue, strongly conserved residues crucial for the catalytic activity of selected L-ASNases are indicated by green, observed key substitutions are marked by red. Amino acid sequences of hyperthermophiles are marked in green boxes; FDA-approved L-ASNases (in processed form) are marked in blue boxes. Multiple sequence alignments were carried out using Clustal version 2.1 [61].

The phylogenetic tree of L-asparaginases also reveals that the enzyme derived from *T. sibiricus* is more closely related to the reported archaeon L-asparaginases (Figure 1a).

Regardless of the source, L-ASNases contain highly conserved amino acid residues, crucial for their catalytic activity [49,54]. In the case of L-ASNase II from *E. coli* (EcAII), widely used in oncohematology, these residues for the processed enzyme (native signal peptide cleaved) are Thr12, Tyr25, Ser58, Gln59, Thr89, Asp90, Lys162 and Glu283 (Figure 1b). According to the amino acid sequence alignments, for TsA, the residue corresponding to Gln59 is substituted by Thr (Figure 1b). It is known that Gln59 is one of the key residues that interact with the amino group of the substrate [49]. At the same time, Gln59 is also not conserved in type I L-ASNases originating from *E. coli*, *R. rubrum* and *B. subtilis*. This indicates that recognition mode for the substrate amino group differs between the type I and type II L-ASNases.

TsA displayed the same conserved residues as L-ASNases originating from *Thermococcus* sp. and *Pyrococcus* sp. (Figure 1b). In previous crystallographic studies on *P. horikoshii* L-asparaginase, eight residues, namely Thr11, Tyr21, Ser52, Thr53, Thr83, Asp84, Lys154 and Lys274, were found to be crucial for the catalytic activity of the thermophilic enzyme [49]. As shown in Figure 1b, these residues are highly conserved in *T. sibiricus* L-ASNase, except the residue corresponding to Lys274 is substituted by glutamic acid as in other L-ASNases originating from *Thermococcu*s sp.

Bansal et al. showed that Lys274Glu mutant of *P. furiosus* L-ASNase (PfA) has improved enzymatic properties in physiological conditions as compared to the wild type. K274E mutant displayed ~2.5-fold higher catalytic efficiency, reduced activation energy and 2-fold lower Km as compared to the wild type at 37 °C [46].

It is considered that such replacements of opposite charge residues in Glu makes the loop more flexible and mobile [43]. Analysis of TsA sequence confirms that this mutation may be characteristic of all type I L-ASNases from *Thermococcus* sp.

### 2.2. Gene Cloning, Expression and Recombinant Enzyme Purification

The native L-asparaginase gene from *T. sibiricus* (*tsA*_wt) was artificially synthesized and cloned into the pET-28a(+) vector under the control of the T7 promoter.

For more efficient heterologous protein expression in *E.coli* cells, synthetic gene *tsA*_mod was engineered using codon optimization (Supplementary Materials, Figure S1). The codon adaptation index (CAI) reached 0.72, close to desired >0.8. After codon modification, the final optimized sequence demonstrated a shift in GC percentage from 38.5% to 50.18%. Synthetic gene *tsA*_mod was also cloned into pET-28a(+) vector.

Constructed plasmids were transformed into the host *E. coli* BL21 (DE3) for the heterogeneous expression of TsA. Analysis of L-asparaginase activity assayed in bacterial lysates of the recombinant strains showed that only synthetic gene (*tsA*_mod) favored heterologous expression and resulted in high yields of active enzyme TsA in host strains. This enzyme was further purified and characterized.

Purification of TsA was achieved by ion-exchange chromatography with a final yield of 78.8% (Table 1).

**Figure 2:**
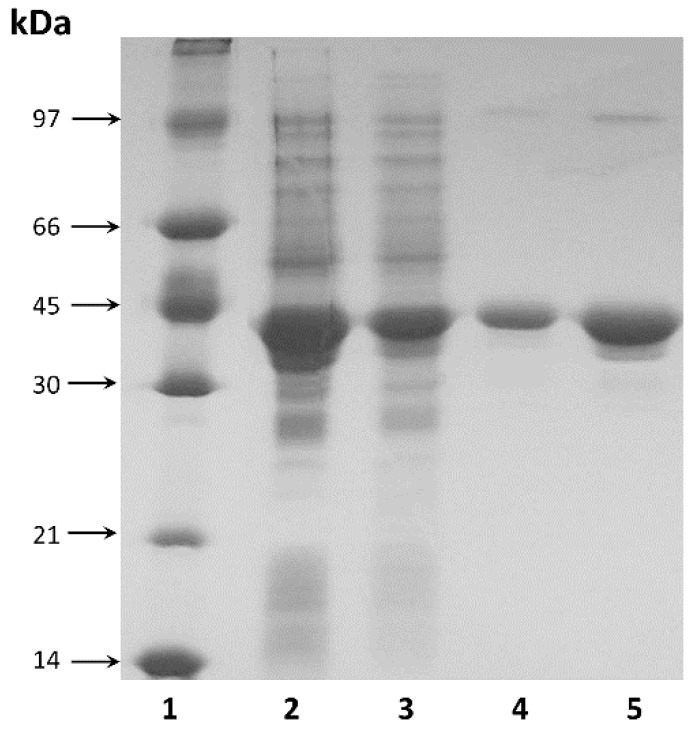
SDS-PAGE analysis: 1—protein molecular weight marker; 2, 3—TsA in cell free homogenate at concentrations 100 µg and 20 µg, respectively; 4, 5—purified TsA at concentrations 2 µg and 10 µg, respectively.

The purified enzyme showed electrophoretic homogeneity with a single major band on SDS-PAGE (Figure 2). The molecular weight of the purified enzyme was estimated to be approximately 37.5 kDa by SDS-PAGE (Figure 2), which was consistent with the theoretical value calculated from the amino acid sequence (36.6 kDa).

### 2.3. Specific Activity of TsA and Enzyme Kinetics

The recombinant L-ASNase exhibited high hydrolysis activity toward L-Asn with specific activity of 2164 U/mg. Substrate specificity experiments demonstrated that TsA has low glutaminase activity: the enzyme could utilize L-glutamine as substrate with approximately 7% of the enzyme activity when L-Asn was used as substrate.

The kinetic properties of the recombinant L-ASNase from *T. sibiricus* were assessed. V_max_ was found to be 1200 µM/min. The K_M_ for substrate L-Asn was estimated to be 2.8 mM.

### 2.4. Effect of Temperature and pH on Enzyme Activity

**Figure 3:**
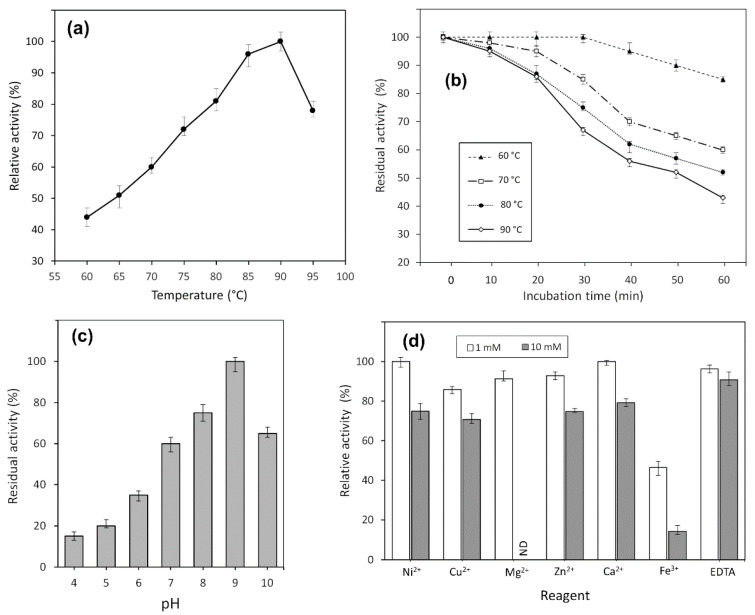
Effect of temperature on (**a**) activity and (**b**) stability of TsA. Stability is expressed as residual activity (%) after 0–60 min of incubation. Effects of (**c**) pH and (**d**) various metal cations on the catalytic activity of the TsA; ND, not defined.

Activity of TsA was evaluated at different temperatures, ranging from 60 to 95 °C. It was shown that the enzyme exhibited its maximum activity at 90 °C but was also active over a wide temperature range (Figure 3a). The time course of thermal inactivation of purified L-asparaginase is shown in Figure 3b.

TsA displayed thermostability with residual activity of more than 80% after 60 min incubation at 60 °C. The enzyme retained 86% of its initial activity at 90 °C after 20 min incubation (Figure 3b).

The enzymatic activity of the purified enzyme was monitored in different pH systems, ranging from pH 4.0 to 10.0, as described in Section 4. The pH-dependent activity profile is given in Figure 3c.

The optimal activity of TsA was detected at pH 9.0, but the recombinant enzyme also exhibited high relative activity in a range of pH 7.0–10.0 independently of buffer system.

### 2.5. Chemical Stability and Effect of Metal Ions on TsA Activity

The recombinant L-ASNase from *T. sibiricus* was remarkably stable in urea solutions. Various concentrations of urea did not result in a significant decrease in enzyme activity until a final concentration of 6 M, reflecting no unfolding of the protein.

The effect of various metal cations Ni^2+^, Cu^2+^, Mg^2+^, Zn^2+^ and Ca^2+^ (1 mmol/L and 10 mmol/L) and EDTA (1 mmol/L and 10 mmol/L) on TsA activity was examined. No significant change in the enzyme activity was observed in the presence of any metal ions or EDTA except for Fe^3+^. There was a 53.5% decrease in enzyme activity in the presence of 1 mmol/L Fe^3+^ (Figure 3d). The addition of Fe^3+^ at 10 mmol/L caused a drop in L-asparaginase activity of more than 85%.

These results indicate that most metal ions had no significant inhibitory effect on the relative TsA enzyme activity. Reduced L-ASNase activity was observed only in the presence of Fe^3+^.

### 2.6. Determination of TsA Cytotoxic Activity

According to experimental data, the activity of TsA under optimal conditions (90 °C and pH 9.0) is 2164 U/mg. At 37 °C and pH 7.1 (in Dulbecco′s phosphate-buffered saline), activity decreases to 224 U/mg, but it is also higher than the optimal activity of 73 U/mg exhibited by L-ASNase II from *E. coli* [62]. Thermophilic L-ASNases can compete with those of mesophilic origin in relatively mild reaction conditions and may be considered as potential anticancer agents.

Here we report that L-ASNase from *T. sibiricus* has strong cytotoxic activity toward cancer cells. To test cytotoxic activity, different types of human cancer cells and normal fibroblasts were cultivated at 37 °C in the presence of different concentrations of the enzyme, and cell viability and apoptosis induction were measured in 72 h of incubation. Table 2 represents IC_50_ and IC_90_ values for tested cell lines.

**Figure 4:**
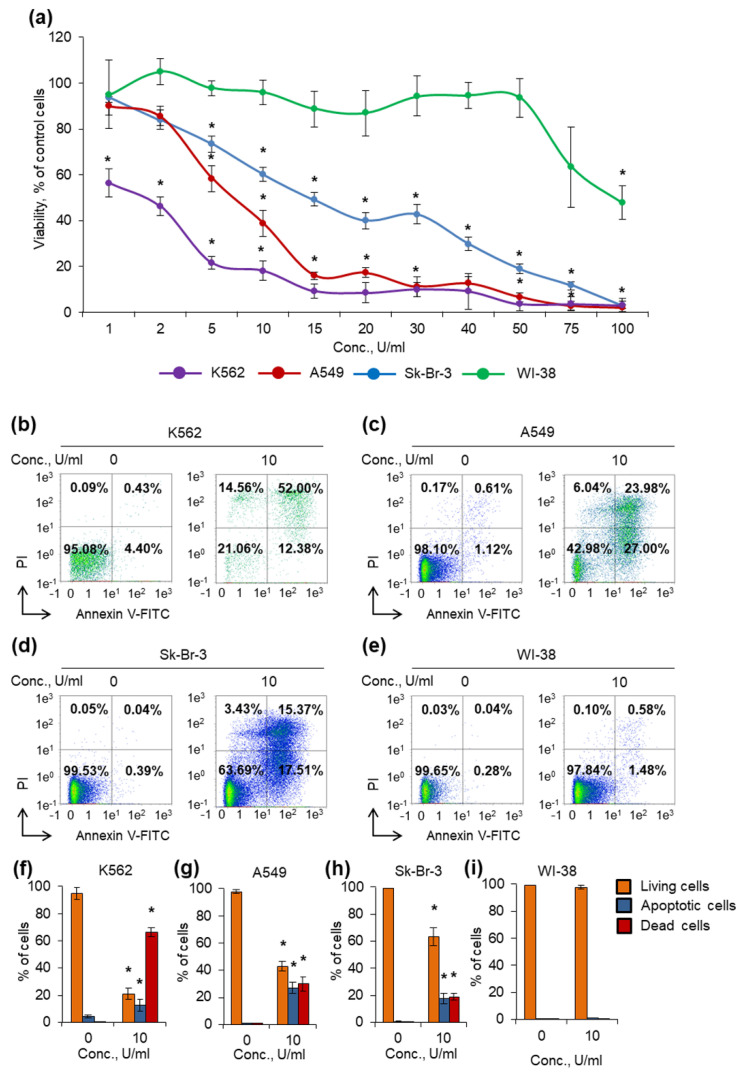
Cytotoxic activity of the enzyme against cancer cells. Cancer cells and normal fibroblasts were cultivated for 72 h in the presence of different concentrations of the enzyme. (**a**) Cell viability measured by MTT test. (**b**–**e**) Representative flow cytometry diagrams for cells incubated with 10 U/mL of the enzyme and labeled with annexin V-FITC and PI. Ratios of living cells (lower left quadrants), apoptotic cells (lower right quadrants) and dead cells (two upper quadrants) are presented. (**f**–**i**) Histograms of live, apoptotic and dead cells incubated with 10 U/mL of the enzyme. Conc., concentration. PI, propidium iodide. *n* = 4. * *p* ≤ 0.05 vs. control untreated cells.

K562 cell line was the most sensitive among cancer cells, and the enzyme was able to decrease cell viability at the lowest concentration, 1 U/mL (Figure 4a). Sk-Br-3 cell line was the most resistant, and a number of cells remained alive even at the concentration of 50 U/mL. A549 cell line demonstrated rather moderate sensitivity for the enzyme among cancer cells. Normal human fibroblast WI-38 cells were almost insensitive, and the enzyme was able to significantly decrease the viability at the highest concentration tested, 100 U/mL.

Different L-ASNases are known to be able to induce cancer cell death by apoptosis [3,63]. We incubated cells with 10 U/mL of the enzyme and measured the induction of apoptosis by labeling phosphatidyl serine on cell membranes with annexin V-FITC and cell DNA by PI followed by flow cytometry. The results of apoptosis measurement were in good agreement with the results from the MTT test. The enzyme induced apoptosis more efficiently in K562 cells: about 20% of cells remained alive after incubation (Figure 4b,f). Sk-Br-3 and A549 cells appeared to be the more resistant: 64% and 43% of cells remained alive after incubation, respectively (Figure 4c,d,g,h). The enzyme did not induce apoptosis in normal fibroblasts at the concentration of 10 U/mL (Figure 4e,i). The results demonstrated that the enzyme has cytotoxic activity and can induce apoptosis in cancer cells, while normal cells are almost unsensitive to its activity (Figure 4, Supplementary Materials Figure S2).

## 3. Discussion

The increasing need for more robust industrial L-ASNases attracts much attention to discovering new potential sources of the enzyme. In this report, we identified and described a novel L-ASNase derived from hyperthermophilic anaerobic archaeon *T. sibiricus*.

The clustering pattern of amino acid sequences represents that putative L-ASNase from *T. sibiricus* is more closely related to the archaeon L-ASNases originating from *Thermococcus* sp. and *Pyrococcus* sp. and shares a low level of sequence identity with mesophilic L-ASNases. Available crystallographic data of L-ASNases of various origins indicate that, despite the low degree of homology, which may not exceed 20–30%, they have conserved regions that determine the similarity of their tertiary structures and the catalytic mechanism [49,54,59,64]. According to the amino acid sequence alignments, TsA displayed eight conserved residues (Figure 1b) essential for the catalytic activity, namely Thr12, Tyr22, Ser55, Thr56, Thr86, Asp87, Lys157 and Glu279.

In this work, for characterization of the novel L-ASNase, we employed codon optimization as a tool to re-engineer the original gene, which enabled us to successfully express it in heterologous host *E. coli* (Supplementary Materials, Figure S1).

The recombinant protein was purified with a yield of about 80% and displayed the specific activity of 2164 U/mg. A summary of biochemical properties of currently characterized L-ASNases derived from hyperthermophilic archaeon is presented in Table 3. According to previously reported results, the enzyme from *T. sibiricus* displayed specific activity similar to that of L-ASNase from *T. kodakarensis* KOD1 (2350 U/mg) [34] and much higher than that of the L-ASNase from *P. furiosus* (550 U/mg) [33] and *P. yayanosii* CH1 (1483.8 U/mg) [45] (Table 3). At the same time, L-ASNase activity of TsA was significantly lower compared with that of *T. zilligii* (5278 U/mg) and *T. gammatolerans* EJ3 (7622 U/mg) [44]. However, it is worth mentioning that L-ASNase from *T. gammatolerans* EJ3 not only displayed the highest ever reported L-asparaginase activity but also showed undesired considerable hydrolysis activity toward L-glutamine (2926 U/mg), significantly reducing the possibility of its further application. For L-ASNase from *T. zilligii*, L-glutaminase activity was not determined [43]. In this view, among the characterized hyperthermophilic (as well as mesophilic) homologs, L-ASNase from *T. sibiricus* may be considered as a potential candidate for further research and application due to high L-ASNase activity in combination with glutaminase activity not exceeding 7%.

The kinetic properties of the recombinant L-ASNase from *T. sibiricus* were assessed. V_max_ was found to be 1200 µM/min. The K_M_ for substrate L-Asn was estimated to be 2.8 mM, which was similar to that for the archaeon L-ASNase from *T. kodakarensis* KOD1 (K_M_ = 2.6 mM) (Table 3) [24]. However, in another study, K_M_ value for *T. kodakarensis* KOD1 L-ASNase was reported to be 5.5 mM [34]. Comparison of K_M_ showed that it is lower for TsA than for the enzymes from *T. zilligii* (K_M_ = 6.08 mM) [43], *T. gammatolerans* EJ3 (K_M_ = 10.0 mM) [44], *P. yayanosii* CH1 (K_M_ = 6.5 mM) [45], *P. furiosus* (K_M_ = 12.1 mM) [33] and *T. aquaticus* T351 (K_M_ = 8.6 mM) [65].

It can be seen that the K_M_ values of L-ASNases from thermophiles are quite high compared to those of the nonthermophiles, indicating a rather low affinity to the substrate for thermophilic homologs (Table 3). However, it is worth mentioning that increased K_M_ values have previously been demonstrated for bacterial and eukaryotic enzymes originating from thermophilic microorganisms. The K_M_ values are higher for thermophilic homologs than mesophilic ones for such enzymes as phosphoglycerate kinase [66], glutamate dehydrogenase [67], alkaline phosphatase [68,69,70], GTPase (TrmE) [71] and glucose-6-phosphate dehydrogenase [72].

As a rule, extremophilic L-ASNases display high K_m_ associated with increased k_kat_. Increased k_kat_ or enzyme efficiency k_kat_/K_M_ can be achieved by lowering activation energy and increasing conformational flexibility. On the other hand, such changes result in reduced substrate affinity and increased K_M_.

It appears that adaptation at high environmental temperatures involves an increase in k_kat_ and K_M_ for thermophilic L-ASNases. This characteristic feature allows optimizing catalytic efficiency by reaching a balance between substrate binding and rate of product release.

Comparison of mesophilic and thermophilic L-ASNase homologs clearly demonstrates that the latter have both higher K_M_ values and higher specific L-ASNase activity.

According to experimental data, the optimum conditions of the recombinant TsA were similar to those found for L-ASNases from other members of *Thermococcus* and *Pyrococcus* spp., which exhibited their highest activities at temperatures of 85–95 °C and in a pH range of 8.0–9.5 (Table 3).

Investigation on metal cation effects on TsA activity indicates that most metal ions have no significant inhibitory effect on the relative TsA enzyme activity. Reduced L-ASNase activity was observed only in the presence of Fe^3+^. In previous data, inhibition of the enzyme activity by addition of Fe^3+^ was reported for L-ASNase from mesophilic *Erwinia carotovora* [73] and mutant forms of *Rhodospirillum rubrum* L-ASNase [58].

The recombinant L-ASNase from *T. sibiricus* is remarkably stable in urea solutions. These results are in good agreement with previously reported experimental data: L-ASNases from *T. gammatolerans* EJ3 [44], *T. kodakaraensis* [34] and *P. furiosus* [46] display high resistance against urea denaturation until final concentrations of 6–8 M. Activities of these enzymes are almost not changed when the denaturant urea concentration is not more than 6 M.

TsA displays thermostability with residual activity of more than 80% after 60 min incubation at 60 °C. The enzyme retains 86% of its initial activity at 90 °C after 20 min incubation. From previous studies, it was concluded that L-ASNase treatment at a temperature of around 80 °C for a short period of time was considerably efficient in reducing acrylamide formation in foods [43]. It seems that the thermal stability of TsA is quite sufficient for pretreatment of products and inhibition of acrylamide formation during the baking and frying process in the food industry.

L-ASNases from extremophilic microorganisms exhibit maximal activity under conditions significantly different from physiological; this expands the possibilities of their use, in particular, in high-temperature processes in the food industry. Nevertheless, due to increased stability, these enzymes can compete with L-ASNases of mesophilic microorganisms in relatively mild reaction conditions.

The activity of TsA under optimal conditions (90 °C and pH 9.0) was estimated to be 2164 U/mg. At 37 °C and pH 7.1, it was 224 U/mg, which is 10.4% of the maximal value. This value exceeds the results obtained for mesophilic EcAII—73 U/mg [62]. Similar results were reported for thermophilic L-ASNases. Activity of L-ASNase from *T. zilligii* at 40 °C decreases to 431 U/mg; under optimal conditions (90 °C and pH 8.5), it is 5278 U/mg (Table 3) [43]. At 37 °C, the enzyme from *T. kodakaraensis* KOD1 showed a specific activity of 94 U/mg (4% of its maximum activity at 85 °C, pH 9.5) which is also higher than the activity of EcAII [34]. Hatanaka et al. have shown that L-ASNase from thermophilic bacterium *S. thermoluteus* subsp. fuscus NBRC 14,270 is better than EcAII in terms of specific activity toward L-Asn at 37 °C: 68.09 U/mg vs. 41.41 U/mg [74]. Overall data indicate that thermophilic L-ASNases with relatively low residual activity under physiological conditions may be considered as potential anticancer agents.

In this study, we attempted to evaluate the anticancer effects of TsA in vitro. According to our data, L-ASNase from *T. sibiricus* displays strong cytotoxic activity toward cancer cell lines. IC_50_ values for K562, A549 and Sk-Br-3 cells were estimated to be 1.5, 6.6 and 15.8 U/mL, respectively. At the same time, normal human fibroblast WI-38 cells were almost unsensitive to the enzyme, with IC_50_ of 97.1 U/mL. The results of this experiment demonstrated that even low specific activity of the enzyme at 37 °C can induce apoptosis in cancer cells, but not in normal cells. We believe that it became possible due to prolong stability of the enzyme in cell culture media, which however remains to be investigated. Such an increase stability along with retained enzymatic activity makes this enzyme an attractive agent for anticancer drug which must be tested in vivo.

It is important to note that L-ASNases of various origins differ in immunogenicity profile. It was shown that EcA lacks allergenic cross-reactivity with EwA [75]. There is a clear need for L-ASNases with increased stability (to improve pharmacokinetics and increase the duration of blood circulation) and reduced immunogenicity compared to FDA-approved ones [41]. Expanding the range of available L-ASNases with different epitope profiles and improved characteristics will successfully complete the induction and postinduction stages of ALL therapy.

The next approach to adopt thermophilic L-ASNases for clinical application is the development of their mesophilic mutants. It was shown that such mutants can combine high stability and specific activity under physiological conditions.

Thus, by comparing the main properties of the novel L-ASNase from *T. sibiricus* with previously described homologs from other hyperthermophiles, TsA seems to be promising object for further mutagenesis in order to obtain mesophilic analogs, which will have biotechnology application in view of its high L-ASNase activity, glutaminase activity not exceeding 7%, promising chemical and thermal stability and strong cytotoxic activity toward cancer cells.

## 4. Materials and Methods

### 4.1. Enzymes and Chemicals

All chemicals used in the experiments were of analytical grade and purchased from Fluka Chemical Corp. (Fluka Chemie GmbH, Buchs, Switzerland), Merck (Merck Millipore, Darmstadt, Germany), Bio-Rad (1000 Alfred Nobel Drive Hercules, CA, USA), Reanal (Reanal Finechemical Private Ltd., Budapest, Hungary), Serva (SERVA Electrophoresis GmbH, Heidelberg, Germany), Paneco (Moscow, Russia) or Reachem (Moscow, Russia). Enzymes were purchased from SibEnzyme (SibEnzyme-M, Moscow, Russia).

Growth media for human cancer cell lines were produced by Gibco (Thermo Fisher Scientific Inc., Waltham, MA, USA). All media were supplemented with fetal bovine serum from Capricorn Scientific (Capricorn Scientific, Ebsdorfergrund, Germany) and 1% sodium pyruvate produced by Paneco (Moscow, Russia).

### 4.2. Strains and Cell Lines

*E. coli* XL1-Blue (Stratagen, La Jolia, CA, USA ) and *E. coli* BL21 (DE3) (Novagen, Madison, WI, USA) were used for plasmid amplification and expression, respectively.

Human mammary gland adenocarcinoma Sk-Br-3, lung epithelial carcinoma A549 and chronic myelogenous leukemia K562 cell lines and normal human fibroblasts WI-38 (all from ATCC, Manassas, VA, USA) were used for evaluation of L-ASNase cytotoxic activity.

### 4.3. Cloning of TsA Coding Sequences

A putative gene predicted to encode L-ASNase TSIB_RS08165 (*tsA*_wt) (GeneID: 8096686, sequence 1510265–1511260 https://www.ncbi.nlm.nih.gov/nuccore/NC_012883.1 (accessed on 28 May 2021), protein GenBank accession No. WP_015849943.1) flanked by the restriction sites *Nhe*I/*Sal*I was artificially synthesized by TWIST Bioscience (Twist Bioscience HQ, San Francisco, CA, USA). The synthesized gene was hydrolyzed and cloned into *Nhe*I/*Sal*I digested vector pET-28a(+) under the control of the T7 promoter.

Synthetic gene *tsA*_mod (GenBank accession No. MW981255) was engineered using codon optimization for more effective heterologous expression in *E. coli* cells. Computational optimization was performed by using original TWIST Bioscience algorithms (Twist Bioscience HQ, San Francisco, CA, USA). Artificial sequence was synthesized by TWIST Bioscience (Twist Bioscience HQ, San Francisco, CA, USA). Plasmid for *tsA*_mod expression was constructed as previously described: optimized TsA coding sequence flanked by the restriction sites *Nhe*I/*Sal*I was hydrolyzed and cloned into *Nhe*I/*Sal*I digested vector pET-28a(+) under the control of the T7 promoter. Constructed vectors were transformed and expressed in *E. coli* BL21 (DE3).

### 4.4. Expression and Purification of Recombinant TsA

The selected recombinant *E. coli* clones were grown as previously described [57,76]. Kanamycin 0.05 mg/mL was added into the medium for the cultivation of cells harboring plasmids. The target protein expression was induced by lactose added to the expressed culture at a density of A600 1.9 to a final concentration of 0.2%. The cells were grown for an additional 17–20 h and pelleted by centrifugation at 4000× *g* for 15 min.

All enzyme purification stages were performed at +4 °C. Three grams of biomass was suspended in buffer (20 mM sodium phosphate buffer pH 7.2, 1 mM glycine, 1 mM EDTA) and destroyed by ultrasound treatment [57]. Cell debris and unbroken cells were removed by centrifugation (35,000× *g*, 30 min). Supernatant, containing the enzyme, was applied to SP-Sepharose column. Protein was eluted with a linear gradient of 0–1.0 M NaCl. Column fractions, containing enzyme (0.46–0.7 M NaCl), were collected. Ultrafiltration, desalting and buffer exchange was performed using Amicon membranes (Millipore, Burlington, MA, USA) as described previously [77]. Samples were frozen and stored at −20 °C.

Protein concentration was determined by the method of Sedmak [78] with bovine serum albumin as the standard. SDS-PAGE was carried out to visualize and determine protein purity as previously described [79].

### 4.5. Enzyme Activity Assay and Determination of Kinetic Parameters of TsA

One unit of L-asparaginase activity is defined as the enzyme amount, releasing 1 μM of ammonia per minute under experimental conditions [29,30]. The reactions were performed at 90 °C in Tris–HCl buffer (0.05 M, pH 9.0). The ammonia content was evaluated by the direct Nesslerization [80,81]. L-glutaminase activity was measured by the same procedure using L-glutamine as substrate. Specific enzyme activity was expressed in U/mg protein.

The kinetic parameters of TsA were determined in Tris–HCl buffer (0.05 M, pH 9.0) containing 20–250 μM of L-asparagine at 90 °C. The observed data were fitted to the Michaelis–Menten equation, and the kinetic constants Km and Vmax were calculated from Lineweaver–Burk plots [57].

### 4.6. Effect of Temperature and pH

The activity at different temperatures and pH was studied for the purified enzyme.

For optimum temperature, the enzyme activity profile was analyzed at different temperatures, ranging from 60 to 95 °C with 5 °C increments. The mixture was assayed in Tris–HCl buffer (0.05 M, pH 9.0).

The thermal stability of L-ASNase from *T. sibiricus* was determined by detecting the residual activity of the enzyme that had been preincubated at different temperatures, ranging from 60 to 90 °C with 10 °C increments, in Tris–HCl buffer (0.05 M, pH 9.0).

The optimum pH was determined by assessing the enzyme activity at different pH at 90 °C in four buffer systems, namely sodium acetate (0.05 M, pH 4.0–6.0), sodium phosphate (0.05 M, pH 6.0–7.0), Tris–HCl buffer (0.05 M, pH 7.0–9.0) and glycine–NaOH buffer (0.05 M, pH 9.0–10.0).

### 4.7. Chemical Denaturation Studies and Effect of Various Metal Ions

Enzyme stability was investigated after 1 h incubation in Tris–HCl buffer (0.05 M, pH 9.0) in the presence of 0–8.0 M urea. The activity of TsA examined at 90 °C in the absence of urea was taken as 100%. The measured activities were compared with the activity of the enzyme without urea addition under the same conditions.

The effects of metal ions on the TsA activity were investigated in the presence of various cations (Ni^2+^, Cu^2+^, Mg^2+^, Zn^2+^, Ca^2+^, Fe^3+^) and EDTA. Salts NiCl_2_, CuSO_4_, MgCl_2_, ZnCl_2_, CaCl_2_, FeCl_3_ and EDTA were added at concentrations of 1 mM or 10 mM. The enzyme activity was assayed at 90 °C and pH 9.0 by adding L-asparagine and the corresponding metal ion(s) or EDTA. The entire procedure was triplicated.

The activity without any metal ion addition was set to 100%. The measured activities were compared with the activity of the enzyme without metal ion or EDTA addition under the same conditions.

### 4.8. Determination of Cytotoxic Activity

Human mammary gland adenocarcinoma Sk-Br-3, lung epithelial carcinoma A549 and chronic myelogenous leukemia K562 cell lines were grown in RPMI-1640 medium. Normal human fibroblasts WI-38 were grown in DMEM medium. All media were supplemented with 5% fetal bovine serum and 1% sodium pyruvate, and cells were grown at 5% CO_2_/95% air in a humidified atmosphere at 37 °C. Cell lines had been tested for mycoplasma contamination before the experiment using Mycoplasma Detection Kit PlasmoTest (InvivoGen, San Diego, CA, USA).

To test acute toxicity, cells were cultivated for 72 h in a 96-well plate (TPP, Trasadingen, Switzerland) in the presence of the enzyme within the range of concentrations 1–100 U/mL, and cell viability was tested by measuring the conversion of the tetrazolium salt, 3-(4,5-dimethyl-thiazol-2-yl)-2,5-diphenyltetrazolium bromide, to formazan (MTT test). IC_50_ and IC_90_ values (the concentration of the enzyme where the response is reduced by 50% and 90%, respectively, were calculated from curve-fitting equations) [82].

To measure apoptosis, incubated cells were resuspended in PBS and incubated with annexin V-FITC and propidium iodide (PI) from a FITC Annexin V/Dead Cell Apoptosis kit (Life Technologies, Carlsbad, CA, USA), according to the manufacturer’s protocol. The counting of 5 × 10^4^ cells at each point was performed by flow cytometry with a MACS Quant Analyzer 10 (Miltenyi Biotec GmbH, Bergisch Gladbach, Germany) as we previously described [83].

### 4.9. Statistical Analysis

The experimental data were expressed as mean value ± standard error calculated from three parallel experiments. The statistical analysis was performed by one-way analysis of variance (ANOVA) using Microsoft Excel (version 2016).

In the measurement of cytotoxic activity, statistical analysis involving the Student’s t-test was implemented with the Statistica software (version 9.0, StatSoft, Tulsa, OK). Differences described by *p* ≤ 0.05 were considered significant. The results are presented as mean ± standard error of the mean (SEM).

## Figures and Tables

**Table 1:** Purification yield of recombinant L-asparaginase from *T. sibiricus* (TsA).

Purification Step	Total Protein, mg	Total Activity, U	Specific Activity, U/mg	Yield, %	Purification Fold
crude enzyme	165.0	86,361.0	523.4	100.0	-
purified enzyme	45.0	68,014.3	2164.0	78.8	4.1

- the purification fold was calculated as the ratio between the specific activity of the purified enzyme and enzyme in crude extract sample.

**Table 2:** IC_50_ and IC_90_ values of TsA for cancer and normal cell lines.

Cell Line	IC_50_, U/mL	IC_90_, U/mL
K562	1.5	13.6
A549	6.6	45.4
Sk-Br-3	15.8	82.1
WI-38	97.1	˃300

**Table 3:** The main biochemical properties and kinetic parameters of L-ASNases derived from thermophilic archaea.

Microorganism	Molecular Weight, kDa	Optimum t, °C	Optimum pH	Hydrolysis of L-Asparagine, U/mg	Hydrolysis of L-Glutamine, U/mg	K_M_, mM	V_max_, μM * min^−1^	Reference
*T. sibiricus*	37.5	90.0	9.0	2164	151	2.801	1200	This study
*Thermococcus kodakarensis* KOD1	37.0	90.0	8.0	978.7	Not determined	2.6	1121	[24]
*T. kodakarensis* KOD1	35.5	85.0	9.5	2350	-	5.5	3300	[34]
*T. zilligii*	36.0	90.0	8.5	5278 ± 32	Not determined	6.08		[43]
*T.gammatolerans* EJ3	36.5	85.0	8.5	7622	+2926	10.0		[44]
*P. yayanosii* CH1	36.1	95.0	8.0	1483.8	Not determined	6.5	2929	[45]
*P.furiosus*	~37.0	~85.0	9.0	550	-	80 °C, pH 8.2 12.1 ± 0.05 37 °C, pH 7.4 8.12 ± 0.3		[33,46]
*Pyrobaculum calidifontis*	32	≥100	6.5		-	4.5 ± 0.4	355 ± 13	[50]
*Archaeoglobus fulgidus*		70.0	9.2		+	37 °C 0.08 70 °C 0.005		[23]

- glutaminase-free L-ASNase, +—enzyme with recorded L-glutaminase activity.

## Data Availability

The data presented in this study are contained within the article.

## Supplementary Materials

The following are available online at https://www.mdpi.com/article/10.3390/ijms22189894/s1.

## References

[1] Lopes A.M., de Oliveira-Nascimento L., Ribeiro A., Tairum C.A., Breyer C.A., de Oliveira M.A., Monteiro G., de Souza-Motta C.M., de Magalhães P.O., Avendaño J.G.F. (2017). Therapeutic l-asparaginase: Upstream, downstream and beyond. Crit. Rev. Biotechnol..

[2] Sajitha S., Vidya J., Varsha K., Binod P., Pandey A. (2015). Cloning and expression of l-asparaginase from E. coli in eukaryotic expression system. Biochem. Eng. J..

[3] Song P., Ye L., Fan J., Li Y., Zeng X., Wang Z., Wang S., Zhang G., Yang P., Cao Z. (2015). Asparaginase induces apoptosis and cytoprotective autophagy in chronic myeloid leukemia cells. Oncotarget.

[4] Chen S.-H. (2015). Asparaginase Therapy in Pediatric Acute Lymphoblastic Leukemia: A Focus on the Mode of Drug Resistance. Pediatr. Neonatol..

[5] Mahajan R.V., Kumar V., Rajendran V., Saran S., Ghosh P.C., Saxena R.K. (2014). Purification and Characterization of a Novel and Robust L-Asparaginase Having Low-Glutaminase Activity from Bacillus licheniformis: In Vitro Evaluation of Anti-Cancerous Properties. PLoS ONE.

[6] Ali U., Naveed M., Ullah A., Ali K., Shah S.A., Fahad S., Mumtaz A.S. (2016). L-asparaginase as a critical component to combat Acute Lymphoblastic Leukaemia (ALL): A novel approach to target ALL. Eur. J. Pharmacol..

[7] Hunger S.P., Lu X., Devidas M., Camitta B.M., Gaynon P.S., Winick N.J., Reaman G.H., Carroll W.L. (2012). Improved Survival for Children and Adolescents With Acute Lymphoblastic Leukemia Between 1990 and 2005: A Report From the Children’s Oncology Group. J. Clin. Oncol..

[8] Möricke A., Zimmermann M., Reiter A., Henze G., Schrauder A., Gadner H., Ludwig W.D., Ritter J., Harbott J., Mann G. (2009). Long-term results of five consecutive trials in childhood acute lymphoblastic leukemia performed by the ALL-BFM study group from 1981 to 2000. Leukemia.

[9] Vrooman L.M., Stevenson K.E., Supko J.G., O’Brien J., Dahlberg S., Asselin B.L., Athale U.H., Clavell L.A., Kelly K.M., Kutok J.L. (2013). Postinduction Dexamethasone and Individualized Dosing of Escherichia Coli L-Asparaginase Each Improve Outcome of Children and Adolescents With Newly Diagnosed Acute Lymphoblastic Leukemia: Results From a Randomized Study—Dana-Farber Cancer Institute ALL Consortium Protocol 00-01. J. Clin. Oncol..

[10] Koprivnikar J., McCloskey J., Faderl S.H. (2017). Safety, efficacy, and clinical utility of asparaginase in the treatment of adult patients with acute lymphoblastic leukemia. Onco. Targets. Ther..

[11] Shrivastava A., Khan A.A., Khurshid M., Kalam A., Jain S.K., Singhal P.K. (2016). Recent developments in l-asparaginase discovery and its potential as anticancer agent. Crit. Rev. Oncol..

[12] Safary A., Moniri R., Hamzeh-Mivehroud M., Dastmalchi S. (2019). Highly efficient novel recombinant L-asparaginase with no glutaminase activity from a new halo-thermotolerant Bacillus strain. BioImpacts.

[13] Duval M., Suciu S., Ferster A., Rialland X., Nelken B., Lutz P., Benoit Y., Robert A., Manel A.-M., Vilmer E. (2002). Comparison of Escherichia coli-asparaginase with Erwinia-asparaginase in the treatment of childhood lymphoid malignancies: Results of a randomized European Organisation for Research and Treatment of Cancer-Children’s Leukemia Group phase 3 trial. Blood.

[14] Chan W.K., Lorenzi P.L., Anishkin A., Purwaha P., Rogers D.M., Sukharev S., Rempe S.B., Weinstein J.N. (2014). The glutaminase activity of l-asparaginase is not required for anticancer activity against ASNS-negative cells. Blood.

[15] Jia M., Xu M., He B., Rao Z. (2013). Cloning, Expression, and Characterization of l-Asparaginase from a Newly Isolated Bacillus subtilis B11–06. J. Agric. Food Chem..

[16] Safary A., Khiavi M.A., Mousavi R., Barar J., Rafi A.M. (2018). Enzyme replacement therapies: What is the best option?. BioImpacts.

[17] Muso-Cachumba J.J., Antunes F.A.F., Peres G.F.D., Brumano L., Santos J., Da Silva S.S. (2016). Current applications and different approaches for microbial l-asparaginase production. Braz. J. Microbiol..

[18] Mahajan R.V., Saran S., Kameswaran K., Kumar V., Saxena R. (2012). Efficient production of l-asparaginase from Bacillus licheniformis with low-glutaminase activity: Optimization, scale up and acrylamide degradation studies. Bioresour. Technol..

[19] National Toxicology Program (2019). Report on Carcinogens.

[20] Verma N., Kumar K., Kaur G., Anand S.E. (2007). Colik-12 Asparaginase-Based Asparagine Biosensor for Leukemia. Artif. Cells Blood Substit. Biotechnol..

[21] Kumar K., Kataria M., Verma N. (2012). Plant asparaginase-based asparagine biosensor for leukemia. Artif. Cells Nanomed. Biotechnol..

[22] Erdogan A., Koytepe S., Ates B., Yilmaz I., Seckin T. (2014). Preparation of the L-Asparaginase-Based Biosensor with Polyimide Membrane Electrode for Monitoring L-Asparagine Levels in Leukemia. Int. J. Polym. Mater..

[23] Li J., Wang J., Bachas L. (2002). Biosensor for Asparagine Using a Thermostable Recombinant Asparaginase from Archaeoglobus fulgidus. Anal. Chem..

[24] Hong S.-J., Lee Y.-H., Khan A.R., Ullah I., Lee C., Park C.K., Shin J.-H. (2014). Cloning, expression, and characterization of thermophilicL-asparaginase fromThermococcus kodakarensisKOD1. J. Basic Microbiol..

[25] Sarquis M.I.D.M., Oliveira E.M.M., Santos A.S., Da Costa G.L. (2004). Production of L-asparaginase by filamentous fungi. Memórias Inst. Oswaldo Cruz.

[26] Baskar G., Aiswarya R. (2018). Overview on mitigation of acrylamide in starchy fried and baked foods. J. Sci. Food Agric..

[27] Brumano L.P., da Silva F.V.S., Costa-Silva T.A., Apolinário A.C., Santos J.H.P.M., Kleingesinds E.K., Monteiro G., Rangel-Yagui C.D.O., Benyahia B., Junior A.P. (2019). Development of L-Asparaginase Biobetters: Current Research Status and Review of the Desirable Quality Profiles. Front. Bioeng. Biotechnol..

[28] Krishnapura P.R., Belur P.D., Subramanya S. (2016). A critical review on properties and applications of microbial l-asparaginases. Crit. Rev. Microbiol..

[29] Frank B.H., Pekar A.H., Veros A.J., Ho P.P. (1970). Crystalline L-asparaginase from Escherichia coli B. II. Physical properties, subunits, and reconstitution behavior. J. Biol. Chem..

[30] Yun M.-K., Nourse A., White S.W., Rock C.O., Heath R.J. (2007). Crystal Structure and Allosteric Regulation of the Cytoplasmic Escherichia colil-Asparaginase I. J. Mol. Biol..

[31] Kumar S., Dasu V.V., Pakshirajan K. (2010). Localization and production of novel l-asparaginase from Pectobacterium carotovorum MTCC 1428. Process. Biochem..

[32] Tollersrud O.K., Aronson N.N. (1989). Purification and characterization of rat liver glycosylasparaginase. Biochem. J..

[33] Bansal S., Gnaneswari D., Mishra P., Kundu B. (2010). Structural stability and functional analysis of L-asparaginase from Pyrococcus furiosus. Biochemistry.

[34] Chohan S.M., Rashid N. (2013). TK1656, a thermostable l-asparaginase from Thermococcus kodakaraensis, exhibiting highest ever reported enzyme activity. J. Biosci. Bioeng..

[35] Elleuche S., Schröder C., Sahm K., Antranikian G. (2014). Extremozymes—Biocatalysts with unique properties from extremophilic microorganisms. Curr. Opin. Biotechnol..

[36] Herbert R.A. (1992). A perspective on the biotechnological potential of extremophiles. Trends Biotechnol..

[37] Ishino S., Ishino Y. (2014). DNA polymerases as useful reagents for biotechnology—The history of developmental research in the field. Front. Microbiol..

[38] Littlechild J.A. (2015). Archaeal Enzymes and Applications in Industrial Biocatalysts. Archaea.

[39] Martins M.B., Carvalho I. (2007). Diketopiperazines: Biological activity and synthesis. Tetrahedron.

[40] Coker J.A. (2016). Extremophiles and biotechnology: Current uses and prospects. F1000Research.

[41] Irwin J. (2020). Overview of extremophiles and their food and medical applications. Physiol. Biotechnol. Asp. Extrem..

[42] Guo J., Coker A.R., Wood S.P., Cooper J.B., Chohan S.M., Rashidc N., Akhtar M. (2017). Structure and function of the thermostable L-asparaginase from Thermococcus kodakarensis. Acta Cryst..

[43] Zuo S., Zhang T., Jiang B., Mu W. (2015). Reduction of acrylamide level through blanching with treatment by an extremely thermostable l-asparaginase during French fries processing. Extremophiles.

[44] Zuo S., Xue D., Zhang T., Jiang B., Mu W. (2014). Biochemical characterization of an extremely thermostable l-asparaginase from Thermococcus gammatolerans EJ3. J. Mol. Catal. B Enzym..

[45] Li X., Zhang X., Xu S., Zhang H., Xu M., Yang T., Wang L., Qian H., Zhang H., Fang H. (2018). Simultaneous cell disruption and semi-quantitative activity assays for high-throughput screening of thermostable L-asparaginases. Sci. Rep..

[46] Bansal S., Srivastava A., Mukherjee G., Pandey R., Verma A.K., Mishra P., Kundu B. (2012). Hyperthermophilic asparaginase mutants with enhanced substrate affinity and antineoplastic activity: Structural insights on their mechanism of action. FASEB J..

[47] Garg D.K., Kundu B. (2017). Hyperthermophilic l -asparaginase bypasses monomeric intermediates during folding to retain cooperativity and avoid amyloid assembly. Arch. Biochem. Biophys..

[48] Garg D.K., Tomar R., Dhoke R.R., Srivastava A., Kundu B. (2015). Domains of Pyrococcus furiosus l-asparaginase fold sequentially and assemble through strong intersubunit associative forces. Extremophiles.

[49] Yao M., Yasutake Y., Morita H., Tanaka I. (2005). Structure of the type IL-asparaginase from the hyperthermophilic archaeonPyrococcus horikoshiiat 2.16 Å resolution. Acta Crystallogr. Sect. D Biol. Crystallogr..

[50] Chohan S.M., Rashid N., Sajed M., Imanaka T. (2019). Pcal_0970: An extremely thermostable l-asparaginase from Pyrobaculum calidifontis with no detectable glutaminase activity. Folia Microbiol..

[51] Miroshnichenko M.L., Hippe H., Stackebrandt E., Kostrikina N.A., Chernyh N.A., Jeanthon C., Nazina T.N., Belyaev S.S., Bonch-Osmolovskaya E.A. (2001). Isolation and characterization of Thermococcus sibiricus sp. nov. from a Western Siberia high-temperature oil reservoir. Extremophiles.

[52] Mardanov A.V., Ravin N.V., Svetlitchnyi V.A., Beletsky A.V., Miroshnichenko M.L., Bonch-Osmolovskaya E.A., Skryabin K.G. (2009). Metabolic Versatility and Indigenous Origin of the Archaeon Thermococcus sibiricus, Isolated from a Siberian Oil Reservoir, as Revealed by Genome Analysis. Appl. Environ. Microbiol..

[53] Mardanov A.V., Ravin N.V., Svetlitchnyi V.A., Beletsky A.V., Miroshnichenko M.L., Bonch-Osmolovskaya E.A., Skryabin K.G. Thermococcus Sibiricus MM 739, Complete Genome. https://www.ncbi.nlm.nih.gov/nuccore/NC_012883.1.

[54] Dumina M.V., Eldarov M.A., Zdanov D.D., Sokolov N.N. (2020). L-Asparaginases of Extremophilic Microorganisms in Biomedicine. Biochem. Suppl. Ser. B Biomed. Chem..

[55] Pourhossein M., Korbekandi H. (2014). Cloning, expression, purification and characterisation of Erwinia carotovora L-asparaginase in Escherichia coli. Adv. Biomed. Res..

[56] Kotzia G.A., Labrou N.E. (2005). Cloning, expression and characterisation of Erwinia carotovoral-asparaginase. J. Biotechnol..

[57] Pokrovskaya M.V., Aleksandrova S.S., Pokrovsky V., Veselovsky A.V., Grishin D.V., Abakumova O.Y., Podobed O.V., Mishin A.A., Zhdanov D.D., Sokolov N.N. (2015). Identification of Functional Regions in the Rhodospirillum rubrum l-Asparaginase by Site-Directed Mutagenesis. Mol. Biotechnol..

[58] Pokrovskaya M., Aleksandrova S., Veselovsky A., Zdanov D., Pokrovsky V., Eldarov M., Grishin D., Gladilina Y., Toropigin I., Sokolov N. (2019). Physical-Chemical Properties of L-asparaginase Mutants from Rhodospirillum Rubrum which Showed Antitelomerase Activity. Biomed. Chem. Res. Methods.

[59] Pechkova E., Fiordoro S., Sokolov N., Pokrovsky V., Pokrovskaya M., Aleksandrova S., Veselovsky L., Bragazzi N., Giannini M., Pellegrino L. (2017). LB Crystallization and Preliminary X-ray Diffraction Analysis of L-Asparaginase from Rhodospirillum rubrum. NanoWorld J..

[60] Kumar S., Stecher G., Li M., Knyaz C., Tamura K. (2018). MEGA X: Molecular Evolutionary Genetics Analysis across Computing Platforms. Mol. Biol. Evol..

[61] Larkin M.A., Blackshields G., Brown N.P., Chenna R., McGettigan P.A., McWilliam H., Valentin F., Wallace I.M., Wilm A., Lopez R. (2007). Clustal W and Clustal X version 2.0. Bioinformatics.

[62] Yoshimoto T., Nishimura H., Saito Y., Sakurai K., Kamisaki Y., Wada H., Sako M., Tsujino G., Inada Y. (1986). Characterization of polyethylene glycol-modified L-asparaginase from Escherichia coli and its application to therapy of leukemia. Jpn. J. Cancer Res..

[63] Zhdanov D.D., Pokrovsky V., Pokrovskaya M.V., Alexandrova S.S., Eldarov M.A., Grishin D.V., Basharov M.M., Gladilina Y., Podobed O.V., Sokolov N.N. (2017). Rhodospirillum rubrum l-asparaginase targets tumor growth by a dual mechanism involving telomerase inhibition. Biochem. Biophys. Res. Commun..

[64] Swain A.L., Jaskolski M., Housset D., Rao J.K., Wlodawer A. (1993). Crystal structure of Escherichia coli L-asparaginase, an enzyme used in cancer therapy. Proc. Natl. Acad. Sci. USA.

[65] Curran M.P., Daniel R.M., Guy G.R., Morgan H.W. (1985). A specific l-asparaginase from Thermus aquaticus. Arch. Biochem. Biophys..

[66] Bentahir M., Feller G., Aittaleb M., Lamotte-Brasseur J., Himri T., Chessa J.-P., Gerday C. (2000). Structural, Kinetic, and Calorimetric Characterization of the Cold-active Phosphoglycerate Kinase from the AntarcticPseudomonas sp. TACII18. J. Biol. Chem..

[67] Thomas T.M., Scopes R.K. (1998). The effects of temperature on the kinetics and stability of mesophilic and thermophilic 3-phosphoglycerate kinases. Biochem. J..

[68] Copeland W.H., Nealon A.D., Rej R. (1985). Effects of temperature on measurement of alkaline phosphatase activity. Clin. Chem..

[69] Abubakar M., Wasagu R., Umar M. (2013). Kinetic Studies of Alkaline Phosphatase from the Liver of Agama agama Lizard. Niger. J. Basic Appl. Sci..

[70] Mahesh M., Guleria N., Rajesh T. (2010). Isolation and characterization of extracellular thermostable alkaline phosphatase enzyme from Bacillus spp.. Int. J. Appl. Biol. Pharm. Technol..

[71] Singh A.K., Pindi P.K., Dube S., Sundareswaran V.R., Shivaji S. (2009). Importance of trmE for Growth of the Psychrophile Pseudomonas syringae at Low Temperatures. Appl. Environ. Microbiol..

[72] Richer H.B., Brewer J., Fahlman G.G., Gibson B., Hansen B.M., Ibata R., Kalirai J.S., Limongi M., Rich R.M., Saviane I. (2002). The Lower Main Sequence and Mass Function of the Globular Cluster Messier 4. Astrophys. J..

[73] Warangkar S.C., Khobragade C.N. (2010). Purification, Characterization, and Effect of Thiol Compounds on Activity of the Erwinia carotovora L-Asparaginase. Enzym. Res..

[74] Hatanaka T., Usuki H., Arima J., Uesugi Y., Yamamoto Y., Kumagai Y., Yamasato A., Mukaihara T. (2011). Extracellular Production and Characterization of Two Streptomyces l-Asparaginases. Appl. Biochem. Biotechnol..

[75] Wang B., Relling M.V., Storm M.C., Woo M.H., Ribeiro R., Pui C.-H., Hak L.J. (2003). Evaluation of immunologic crossreaction of antiasparaginase antibodies in acute lymphoblastic leukemia (ALL) and lymphoma patients. Leukemia.

[76] Pokrovskaya M.V., Pokrovskiy V.S., Aleksandrova S.S., Anisimova N., Andrianov R.M., Treschalina E.M., Ponomarev G.V., Sokolov N.N. (2012). Recombinant intracellular Rhodospirillum rubrum L-asparaginase with low L-glutaminase activity and antiproliferative effect. Biochem. Suppl. Ser. B Biomed. Chem..

[77] Borisova A.A., El’Darov A.M., Zhgun A.A., Aleksandrova S.S., Omel’Ianiuk N.M., Sokov B.N., Berezov T.T., Sokolov N.N. (2005). Purification and properties of recombinant Erwinia carotovora L-asparaginase expressed in E.coli cells. Biomeditsinskaya Khimiya.

[78] Sedmak J., Grossberg S.E. (1977). A rapid, sensitive, and versatile assay for protein using Coomassie brilliant blue G250. Anal. Biochem..

[79] Laemmli U.K. (1970). Cleavage of Structural Proteins during the Assembly of the Head of Bacteriophage T4. Nature.

[80] Wriston J.C., Yellin T.O. (2006). L-Asparaginase: A Review. Advances in Enzymology—And Related Areas of Molecular Biology.

[81] Wade H.E., Robinson H.K., Phillips B.W. (1971). Asparaginase and Glutaminase Activities of Bacteria. J. Gen. Microbiol..

[82] Denizot F., Lang R. (1986). Rapid colorimetric assay for cell growth and survival. Modifications to the tetrazolium dye procedure giving improved sensitivity and reliability. J. Immunol. Methods.

[83] Zhdanov D.D., Pokrovsky V.S., Pokrovskaya M.V., Alexandrova S.S., Eldarov M.A., Grishin D.V., Basharov M.M., Gladilina Y.A., Podobed O.V., Sokolov N.N. (2017). Inhibition of telomerase activity and induction of apoptosis by Rhodospirillum rubrum L-asparaginase in cancer Jurkat cell line and normal human CD4+ T lymphocytes. Cancer Med..

