# Supplementary material for: A Novel L-Asparaginase from Hyperthermophilic Archaeon Thermococcus sibiricus: Heterologous Expression and Characterization for Biotechnology Application
Source: Int J Mol Sci. 2021 Sep 13;22(18):9894. doi: 10.3390/ijms22189894 (PMC8470970; doi:10.3390/ijms22189894)

Supplementary Material

Figure S1. Sequence alignment of wild-type *ts*A_wt and codon-optimized *ts*A_mod genes

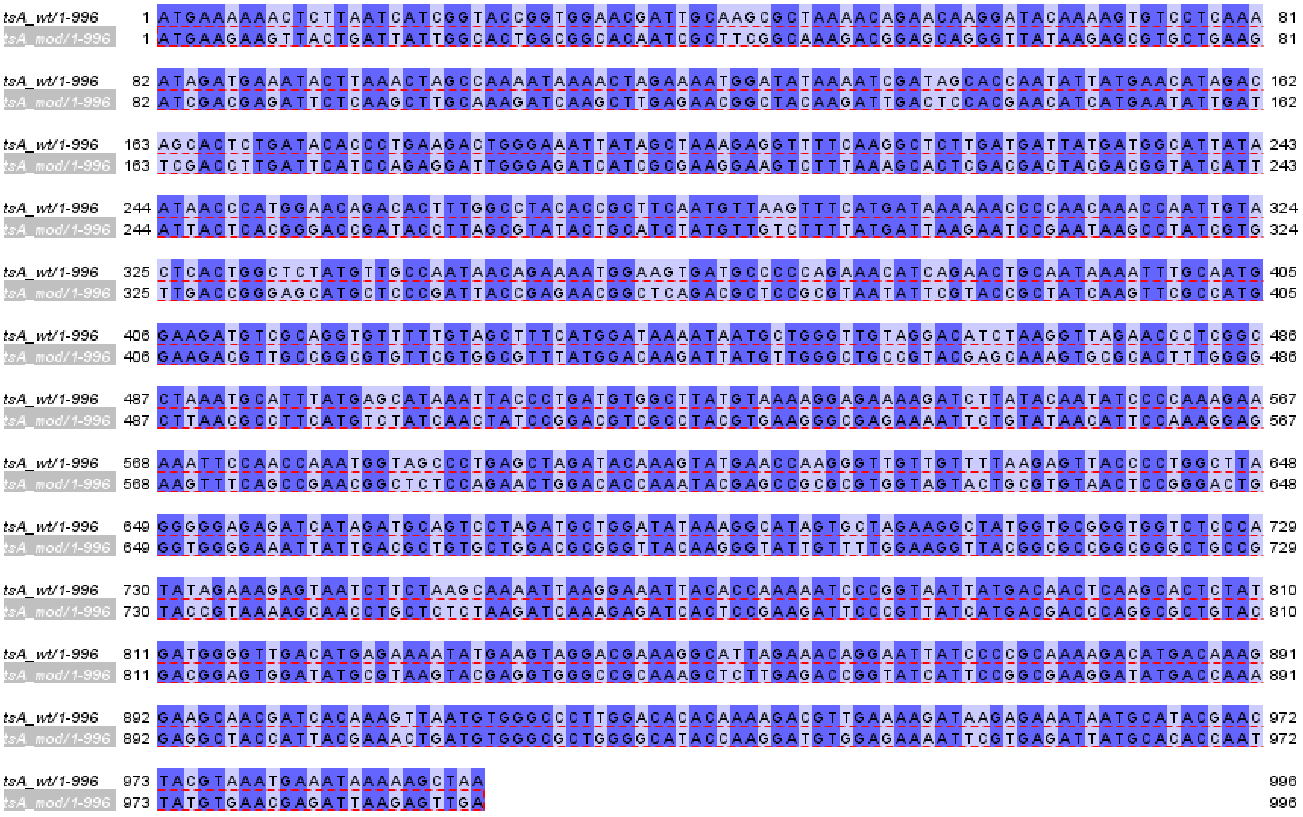

Figure S2. Cytotoxic activity of TsA against cancer cells. Cancer cells and normal fibroblasts were cultivated for 72 h in the presence of different concentrations of the enzyme.

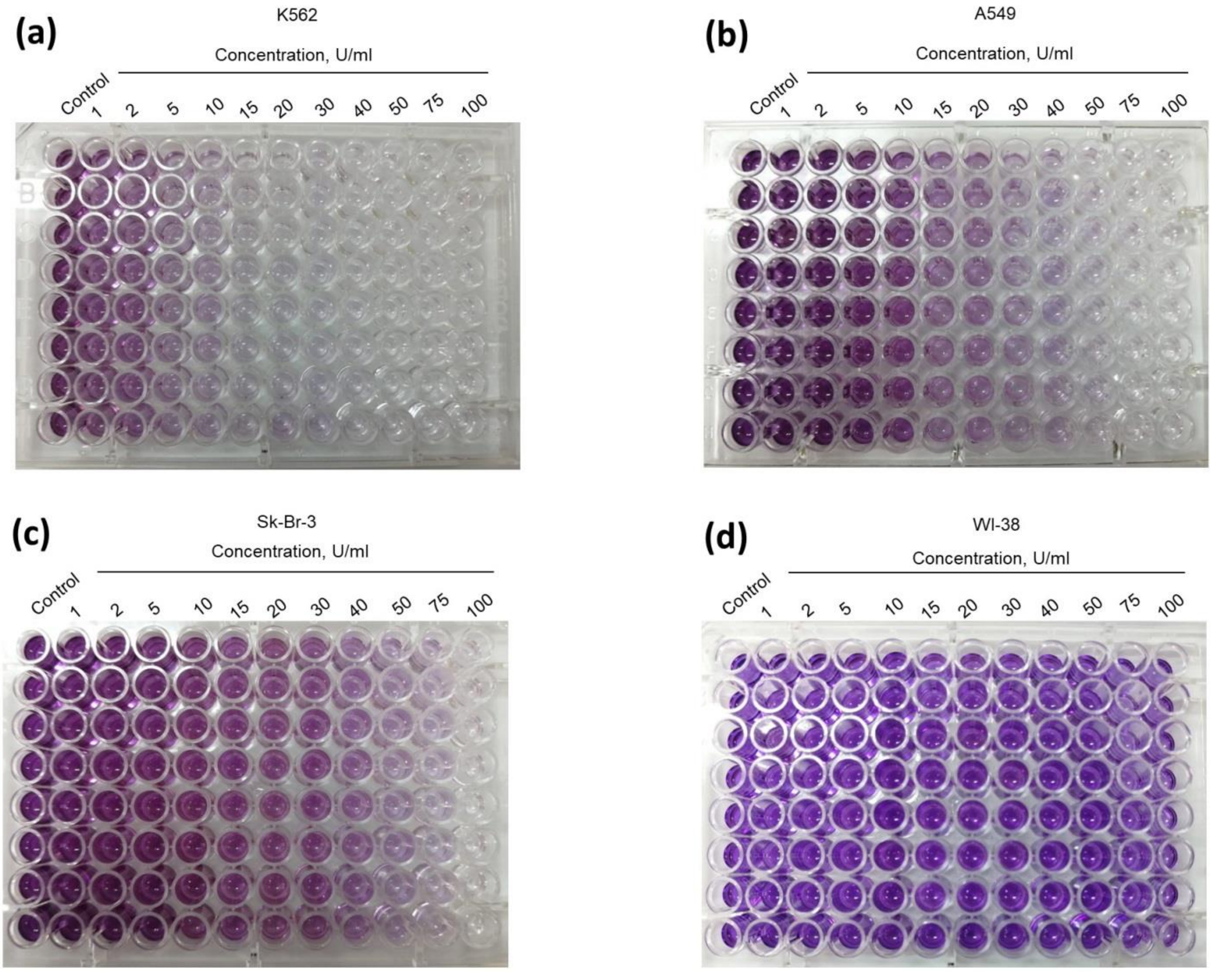

Supplement: Supplementary file 1 [file ijms-22-09894-s001.zip › ijms-1305256-supplementary.pdf]
